# Passive-Sampler-Based Bioavailability Assessment of PCB Congeners Associated with Aroclor-Containing Paint Chips in the Presence of Sediment

**DOI:** 10.1007/s00244-021-00907-2

**Published:** 2021-12-17

**Authors:** Guilherme R. Lotufo, Philip T. Gidley, Andrew D. McQueen, David W. Moore, Deborah A. Edwards, Jeffery Hardenstine, Allen D. Uhler

**Affiliations:** 1 US Army Engineer Research and Development Center, Vicksburg, MS USA; 2 ExxonMobil Environmental and Property Solutions Company, Spring, TX 77389 USA; 3 NewFields-Environmental Forensics Practice, Rockland, MA USA

## Abstract

This is the first investigation of the bioavailability of PCBs associated with paint chips (PC) dispersed in sediment. Bioavailability of PCB-containing PC in sediment was measured using ex situ polyethylene passive samplers (PS) and compared to that of PCBs from field-collected sediments. PC were mixed in freshwater sediment from a relatively uncontaminated site with no known PCB contamination sources and from a contaminated site with non-paint PCB sources. PC < 0.045 mm generated concentrations in the PS over one order of magnitude higher than coarser chips. The bioavailable fraction was represented by the polymer-sediment accumulation factor (PSAF), defined as the ratio of the PCB concentrations in the PS and organic carbon normalized sediment. The PSAF was similar for both field sediments. The PSAFs for the field sediments were ~ 50–60 and ~ 5 times higher than for the relatively uncontaminated sediment amended with PC for the size fractions 0.25–0.3 mm and < 0.045 mm, respectively. These results indicate much lower bioavailability for PCBs associated with PC compared to PCBs associated with field-collected sediment. Such information is essential for risk assessment and remediation decision-making for sites where contamination from non-paint PCBs sources is co-located with PCB PC.

**Supplementary Information:**

The online version contains supplementary material available at 10.1007/s00244-021-00907-2.

PCBs are a class of synthetic chlorinated organic compounds that, due to their chemical and physical stability, were manufactured in the USA under the trade name “Aroclor” between 1929 and 1977 (Miller 1983). Historically, Aroclor mixtures were primarily used in electrical equipment (Miller 1983) but were also widely used as plasticizers within construction materials, including an additive to paint and surface coatings. Chemical-resistant chlorinated rubber paints contained various Aroclors typically at 10–12% (dry weight) of the total composition (Scott and Snyder 2015). Construction materials containing PCBs were widely used in industrial and institutional settings (Scott and Snyder 2015; Jartun et al. 2009), but also in construction and maintenance of military and civilian vessels. PCBs were added to ship paints to give the paints better adhesive properties and to provide protection from corrosion, chemicals and flames (Jensen et al. 1972; Martin and Richards 2010). Aroclor 1254 was the most common plasticizer added to paints through the early 1970s (Scott and Snyder 2015). Additional information on the historic use of PCB-containing paint is provided in the Supplementary Materials.

Vessel maintenance activities, including power washing of vessels and removal of old paint (including paints containing PCBs) via sand blasting, have been identified as a source of PCB contamination in aquatic environments (Jensen et al. 1972; Johnsen and Engøy 2000; Gold and Bloom 2000; Martin and Richards 2010; Bellucci et al. 2016; Oregon Department of Environmental Quality 2020). For example, release of paint residues from ship repair yards and direct release from the hull during port calls were responsible for a considerable fraction of contaminants, including PCBs, associated with sediments outside of ship repair yards and around quay structures in Norway (Johnson and Engøy 2000). In addition to vessel paint, PCB-containing paint from buildings and other structures was found to be the source of PCB contamination of aquatic (Ruus et al. 2006; Jartun et al. 2009) and terrestrial environments (Andersen et al. 2000; Martin and Richards 2010). Bioaccumulation of PCBs in aquatic biota has been attributed to environmental contamination of PCB-containing paint (Jensen et al. 1972; Ruus et al. 2006). Additional information on the environmental contamination by PCB-containing paint, including concentrations reported for biota, sediment and soil at impacted sites are provided in the Supplementary Materials. Regardless of the evidence for the unintended release of PCB-containing paint chips into aquatic environments, only one published study investigated fate and behavior (*i.e.*, leaching; Uhler et al. 2021s), while no known study directly investigated bioavailability or potential for benthic bioaccumulation for paint-associated PCBs.

Due to their hydrophobic nature, PCBs entering aquatic environments strongly adsorb to suspended and bottom sediments. In most contaminated sites, the originating source of PCBs is not co-located with the contaminated sediment. For example, wastewater containing PCBs was historically discharged into the Hudson River (NY, USA) from capacitor manufacturing facilities. At other contaminated sites, materials containing PCBs such as paint, plaster, and caulk remain dispersed in sediment or soil (Andersson et al. 2004; Ruus et al. 2006; Herrick et al. 2007; Martin and Richards 2010; Davies and Delistraty 2016). For sites where PCB-containing manufactured materials such as PCB-containing paint chips (PC) are present, sediment is typically contaminated with PCBs from other sources, such as off-site input. Therefore, understanding the bioavailability for PCBs associated with PC relative to the bioavailability of native sediment PCBs (*i.e.*, differential bioavailability) is greatly desirable.

Bulk sediment chemical concentrations (*C*_total_) have historically been used in contaminated sediment site characterization, risk assessment, and risk management (Greenberg et al. 2014). However, risk assessments and, consequently, risk management decisions and actions carry a relatively high level of uncertainty without an understanding of bioavailability. Passive samplers (PS) of various types have been used as tools to directly sample porewater dissolved hydrophobic organic compounds (HOCs) and reliably estimate freely dissolved concentrations (*C*_free_) in water or porewater (Jonker et al. 2020), which in turn has been shown to be a good thermodynamic metric for bioavailability assessment (Adams et al. 2007; Gschwend et al. 2011; Booij et al. 2016; Smedes et al. 2017; Beckingham and Ghosh 2017; Endo et al. 2020). Passive sampler hydrophobic organic compound (HOC) uptake has also been used as a surrogate for whole-body bioaccumulation in aquatic organisms, in both laboratory and field exposures, as reviewed in Joyce et al. (2016) and Schmidt and Burgess (2020). Therefore, the use of *C*_free_ instead of bulk chemical analyses ensures more certainty for contaminated sediment assessment and risk management (Mayer et al. 2014).

The primary objective of this study was to test the hypothesis of no difference in bioavailability between PCBs associated with PC dispersed in sediments and PCBs associated with field-collected sediments historically contaminated by sources other than paint. Differential bioavailability assessment provides a relative scaling of the tendency for contaminants associated with different environmental media (*e.g.*, different types of organic matter or different manufactured materials), to be strongly sequestered, or quickly released and accumulated by organisms (Beckingham and Ghosh 2017). Investigation of differential bioavailability is facilitated by the use of PS (a uniform phase) which provide bioavailability measurements without the need to account for complex partitioning to a multitude of matrices contained in sediments.

## Material and Methods

### PCB-containing Paint

The chlorinated rubber marine base paint used in this bioavailability experiment, prepared as described in Uhler et al. (2021) using a 1960s-era formula for preparing Aroclor 1254-amended chlorinated rubber marine paint formula, made up to a concentration of approximately 2% Aroclor 1254 (w/w, liquid), which is equivalent to 4% Aroclor 1254 on a dry paint basis. This 2% formulation was used in order to optimize the concentration of PCB in sediment with a maximum density (mass and number) of PC, thereby improving PC homogeneity among experimental sediment treatments. The formulated paint was applied to a steel panel, dynamically aged in seawater for one month following methods described in Kojima et al. (2016), air-dried, and removed using a razor blade (Uhler et al. 2021).

### Preparation of Paint Chip Size Classes

Flakes of PCB-containing paint were ground using a mortar and pestle and dry sieved using stainless steel sieves to achieve three separate PC size classes (“coarse” = 2–5 mm; ASTM #10; “medium” = 0.250–0.300 mm; ASTM #50–60; “fine” =  < 0.045 mm; ASTM #325; Supplementary Materials Fig. S1). Following grinding, the PC were stored in amber glass jars at 4 °C prior to addition to sediment for experimental purposes.

Due to the role of surface area in chemical reactivity (*e.g.*, sorption and desorption), both specific surface area and surface-area-to-volume ratios were calculated for the three size classes of PC. For the “coarse” PC, the specific surface area (cm^2^/g) was estimated based on the geometry of a flat (plate-like) structure (Eq. 1; Pennell 2005).

1 $${\text{Specific surface area }}\left( {\text{flat stucture}} \right) = \left( {2ab} \right)/\left( {\rho V} \right)$$

where ρ is the density of PC (῀2.8 g/cm^3^ for dry 4% Aroclor 1254 PC), *a* is the length, *b* is the width, and *V* is the volume (*i.e.*, length (*a*)*width (*b*)*height (*c*)) of the structure. Length (*a*) and width (*b*) estimates were based on sieve diameter sizes. The average thickness (*c*) of the dried PC was approximately 0.078 mm.

For the ground PC medium and fine size classes, the particle shape was assumed to approximate a sphere, where the specific surface area (cm^2^/g) is estimated using Eq. 2 (Pennell 2005).

2 $${\text{Specific surface area }}\left( {{\text{sphere}}} \right) = 3/\left( {\rho r} \right)$$

where *ρ* is the density of PC (῀2.8 g/cm^3^), and *r* is the radius of the particle (based on sieve equivalent diameter sizes).

Estimates of surface area-to-volume (*S*:*V*) ratios were 1,333, ῀220, and ῀250 for the fine, medium and coarse fractions, respectively (Table 1). It should be noted that these values are estimates as they do not account for surface texture, non-spherical particles, or finer particles that may be present (Pennell 2005).

**Table 1:** Specific surface area and *S*:*V* estimations for paint particles

Size class	Diameter (mm)	Estimated specific surface area^a^ (cm^2^/g)	S:V
Coarse	2–5	92^a^	256^c^
Medium	0.25–0.3	71–86^b^	200–240^d^
Fine	0.045	476^b^	1,333^d^

^a^ Based on Eq. (1); ^b^ based on Eq. (2), ^c^*S*:*V* = 2(ab)/V, ^d^*S*:*V* = [4πr^2^]/[(4/3)πr^3^] or 3/r

### Sediments

Sediment with relatively low levels of PCBs was collected from a single location at Horseshoe Lake (HSL) a pristine oxbow lake alongside the Mississippi River (Warren County, Mississippi, USA), with no known sources of PCBs and low levels of other pollutants (Supplementary Materials). The HSL sediment was predominantly fine-grained (94% silt and clay, 6% sand) and the organic carbon (OC) content was 3.6%. The sediment was thoroughly homogenized with a propeller mixer (Lightnin® Vari-Mix portable mixer; Mixing Equipment) and stored at 4 °C in clean polyurethane buckets before use. The concentration of ∑PCBs (sum of 33 detected congeners) was 0.013 mg/kg dry wt. Detailed analytical data are provided in the Supplementary Materials.

Sediment contaminated with relatively high concentration of PCBs was collected from several locations in the Manistique Harbor (MH) Superfund site located in Manistique, Michigan on the southern shore of Michigan’s Upper Peninsula. The primary sources of contamination at this site include release of PCBs from former paper mill and lumber mill operations, discharge from area industrial facilities and nonpoint sources. Fish collected at the site have elevated levels of PCBs, indicating bioavailability and bioaccumulation of contaminants from the sediment (Gustavson 2014). The field-collected sediments were thoroughly homogenized with a propeller mixer and stored at 4 °C in clean polyurethane buckets before use. MH sediment was predominantly fine-grained (73% silt and clay, 27% sand). The average concentration of ∑PCBs sum of 171 detected congeners and OC content were 5.84 ± 1.78 mg/kg and 6.7 ± 0.3% (*n* = 4). Detailed analytical data are provided in the Supplementary Materials.

### Ex Situ Passive Sampling

Each PS consisted of a 2.5 cm X 6 cm (~ 25 mg) PE (17.2 μm thick, HDX™ brand) coupon. The PS were cleaned by soaking at least three times in dichloromethane (DCM). Each soaking lasted 2 to 3 days. Clean DCM was used for each cleaning cycle. The PS were then rinsed with water multiple times, each for a period of days. Each time, clean Milli-Q water was used. The PS were stored in water (sealed in a jar) for approximately 8 weeks prior to use.

Sediment was portioned into approximately half-liter glass jars (16-oz) with Teflon lined caps. One PS was then added to each jar. PC were then added to create mixtures of sediment, PC and one PS. Each mixture was a fluid slurry, and at least 25% of the volume of the jar was left as headspace. The mass of the PE coupon corresponded to approximately 0.01% of the sediment mass, and depletion was expected to be minimal and acceptable according to criterion in Jonker et al. (2020). Hand shaking of the jars confirmed that the slurries would move inside the jars during mixing. The jars were rotated end-over-end at 30 revolutions per minute (rpm) most of the time. However, initially, not all jars could fit on the end-over-end mixer, so some jars were placed on a slower mixer at 5 rpm (end-over-end). The jars were rotated across mixers, so that all jars spent at least two thirds of their mixing time at 30 rpm. Rotation was accomplished by removing jars from the mixers, hand shaking the jars, and placing them back on the mixers in different locations/configurations. The aim was to promote and maximize a well-mixed slurry environment in the jars for the longest time possible within the timeframe of the project. At the termination of the mixing period, PS were retrieved from the jars using tweezers. The coupons were rinsed with deionized water and wiped with a lint-free paper tissue to remove sediment particles from the surface of the PS. The PS were individually tightly wrapped in aluminum foil. Each wrapped PS was placed in a 40-mL amber glass vials with Teflon lids. A few drops of water were placed on the vials to maintain moisture (Apell and Gschwend 2016) to lessen loss of PCBs from the PS. The water did not come in contact with the PS. The vials along with the sediment recovered from each jar were shipped on ice overnight to Alpha Analytical Laboratory (Mansfield, MA, USA) for PCB analysis.

### Bioavailability of Paint-Associated PCBs in the Presence of Sediment: Effect of Paint Chip Size

To evaluate the effect of °ilability, four ex situ passive sampling jars were set up for each PC size class. Each jar received 241.5 ± 0.2 g wet weight of the HSL sediment corresponding to 83 g of dry sediment, one PS and 10 ± 0.1 mg of PC targeting 4 mg/kg ∑PCBs as PC to create the class size treatments HSL + PC_FINE_, HSL + PC_MEDIUM_, and HSL + PC_COARSE_. This concentration was selected to match the ∑PCBs concentration in the MH sediment according to historic data and was approximately four times higher than high-end concentrations reported for sites impacted by PCB-containing paint ( Supplementary Materials). For ∑PCBs in the bulk sediment, the concentration of PC PCBs exceeded the concentration of native PCBs the sediment by 308-fold. The contents of the jars (sediment, PC, and PS) were mixed for 60 d to allow for PCB redistribution between PC, sediment and PS as described in “*Ex situ* passive sampling.” A time period of 60 d was previously reported as sufficient time for sediment-associated PCBs to attain thermodynamic equilibrium with 25-µm PS during ex situ passive sampling (Lohmann et al. 2012; Apell and Gschwend 2016). To verify if equilibrium of PCBs was achieved between sediment and PC, and PS, additional jars were set up as described above but only for HSL + PC_FINE_ targeting 4 mg/kg ∑PCBs as PC and mixed for 119 and 158 d, four jars per time point. Even though the concentrations of native PCBs in HSL sediment were low, they were above detection limit for 33 congeners. Therefore, HSL sediment without added PC was also evaluated for bioavailability using PS as described above. A summary description of the above treatments is provided in Table S1.

### Bioavailability Evaluation Using an Historically Contaminated Sediment Amended with Paint Chips

For this component of the investigation, two treatments for ex situ passive sampling with PE PS were set up as described above: 1) MH sediment only, and 2) MH sediment and the fine PC fraction combined (MH + PC_FINE_). The primary goal was to conduct a bioavailability comparison for paint-associated PCBs and sediment-associated PCBs. A summary description of the above treatments is provided in Table S1.

For this experiment, each jar received 268 ± 0.2 g wet weight MH sediment corresponding to 92 g of dry sediment and one PS. The MH + PC_FINE_ treatment was created by adding 10 ± 0.1 mg of the fine fraction of PC to MH sediment targeting 4 mg/kg ∑PCBs as PC (target total PCBs concentration = 9.8 mg/kg dry wt.). Four jars were set up for each treatment and the contents of the jars (sediment, PC, and PS) were mixed for 60 d. The average concentration of ∑PCBs (sum of 180 detected congeners) for the MH + PC_FINE_ treatment, based on sediments obtained from replicate jars after ex situ passive sampling, was 8.25 ± 0.74 mg/kg.

#### Chemical Analysis

*Sediment extraction* Approximately 20 g of well homogenized sediment was weighed into a Teflon™ extraction jar, dried with sodium sulfate, fortified with ^13^C-labled PCB congener surrogates (^13^C-PCB 19 and ^13^C-PCB 202), and serially extracted three times with DCM using an end-over-end mixer. Extracts were filtered through glass wool containing sodium sulfate and concentrated on a hot water bath. Extracts were cleaned with activated copper to remove sulfur and processed through a 5 g activated silica gel column following USEPA Method 3630C (USEPA 1996a). Extracts were further cleaned by high pressure liquid chromatography (HPLC) containing a size exclusion gel permeation column (GPC) following USEPA Method 3630A (USEPA 1996b). Extracts were solvent exchanged into hexane and cleaned using concentrated sulfuric acid following EPA Method 3665 (USEPA 1996c). Individual reporting limits were calculated for each sample and ranged from 0.15 to 0.48 µg/kg. Detailed information is provided in the Supplementary Materials.

*PS extraction* Each PS was placed into a 250 mL amber glass jar equipped with a Teflon™ liner, dried with sodium sulfate, fortified with the surrogate compounds listed above, and serially extracted three times with DCM using a shaker table. Extracts were filtered through glass wool containing sodium sulfate and concentrated on a hot water bath. Extracts were cleaned with activated copper to remove sulfur, solvent exchanged into hexane and acid cleaned using sulfuric acid. Individual reporting limit was 0.5 ng. Detailed information is provided in the Supplementary Materials. *Instrumental analysis*. All extracts were fortified with an internal standard and analyzed using an Agilent HP6890 or equivalent equipped with a Restek RTX-PCB 60-m × 0.18 mm ID, 0.18 um film thickness, fused-silica capillary column and a mass spectrometer operated in the selected ion monitoring mode (SIM). The concentrations of 207 individual congeners (some co-eluted) were quantified versus internal (*i.e.*, injection standards) standards, which were spiked into the sample extract prior to analysis. The target congener concentrations were quantified using average response factors generated from a minimum of a 6-point multi-level calibration curve. Sample extracts were analyzed for 209 PCB congeners using USEPA Method 680 (Stevens et al. 1985). Sample-specific surrogate recovery data for these analyses are compiled in the Supplementary Materials. Surrogate recoveries ranged from 60 to 96% with an average of 79%.

*Total Organic Carbon (TOC)* Approximately 10 mg of sample (pre-treated with 10% hydrochloric acid, dried, and homogenized) was weighed into a tin capsule and analyzed using a CHNS/O Analyzer for TOC per USEPA 9060 (USEPA 2004). All analysis was performed in duplicate, and the average TOC value was reported.

#### Quality Control

A series of quality control samples were included to monitor laboratory contamination, extraction efficiency, and reproducibility. This was accomplished through the use of procedural blanks, laboratory control samples/duplicates (LCS/LCSD), surrogates, laboratory duplicates, and National Institute of Standard Reference Material (NIST SRM).

No analyte was detected in the procedural blanks above the reporting limit. All laboratory spiked surrogates and LCS compounds met data quality objective (DQO) recoveries (50–125% and 40–140%, respectively). The laboratory duplicates had a relative percent difference (RPD) < 30% for over 90% of the analytes detected above the reporting limit, and the NIST SRMs met the laboratory DQO recoveries (40–140%) for all certified analytes detected above the reporting limit (see Supplementary Materials). Detailed information on quality control and quality assurance is provided in the Supplementary Materials.

#### Data Analysis

To best illustrate differences in bioavailability relative to bulk sediment concentrations (*C*_total_) among treatments, PCB concentrations in the PS were divided by the OC-normalized bulk PCB concentration in the sediment treatment. The resulting ratio is here referred to as polymer-sediment accumulation factor (PSAF).

3 $${\text{PSAF = }}\frac{{\text{Concentration in the PS}}}{{{\text{Concentration in sediment }}\left( {\text{OC - normalized}} \right)}}$$

The PSAF is well suited for illustrating differences in potential bioavailability among sediments with widely varying *C*_total_ and sediment OC content.

Statistical comparisons were performed using SigmaStat v3.5 software (SSPS, Chicago, IL, USA). Normality was confirmed by the Shapiro–Wilk test, and equal variance was confirmed using the Brown–Forsythe test. One-way ANOVA was performed to determine statistically significant differences (*α* = 0.05) across three or more treatments. The Holm-Sidak method was employed for pairwise multiple comparisons to determine statistical significance between treatments. When assumptions of parametric ANOVA were not met, the data were log-transformed. The nonparametric Kruskal–Wallis one-way ANOVA on ranks was applied when assumptions of parametric ANOVA were not met for log-transformed data. The Dunn’s method was employed for pairwise multiple comparisons to determine statistical significance between treatments. The Student’s t test was used to determine whether statistically significant differences existed between treatment groups (*α* = 0.05). When assumptions of parametric t test were not met, the nonparametric Mann–Whitney Rank Sum test was applied.

## Results

### Bioavailability of paint-associated PCBs dispersed in HSL sediment

The congener profile for solvent-extracted PC mechanically reduced to a size that would pass through a 9.5 mm standard sieve (Uhler et al. in 2021) and solvent extracted HSL + PC_FINE_ was compared. The congener profile is presented here as the relative contribution of PCB congeners to the ∑PCB concentration, which was similar for a subsample of the PC used in this study and PC mixed with HSL sediment for 60 d (*i.e.*, the HSL + PC_FINE_ treatment) (Supplementary Materials Fig. S2). The ratio of PC and HSL + PC_FINE_ ranged from 0.83 to 1.38 (average = 1.01). This indicates that the mixing of PC with sediment did not cause significant changes in the relative concentration of the congeners and the congener profile of the HSL + PC_FINE_ was representative of the PC profile, despite low levels of PCBs in the HSL sediment. For Supplementary Materials Fig. S2 and other figures showing congener data for HSL + PC treatments, a subset of analytes was selected for illustration of congener-specific trends in lieu of showing all data for congeners detected in sediment and PS. The individual analytes shown in Supplementary Materials Fig. S2 (and also in Figs. 2, and Supplementary Materials Figs. S3, S4 and S5) had a contribution to the total concentration in the PC of 0.5% or more. PCBs 84, 89 and 92 were omitted because of analytical interferences on the chromatograms. Detailed analytical data are provided in the Supplementary Materials.

**Fig. 1:**
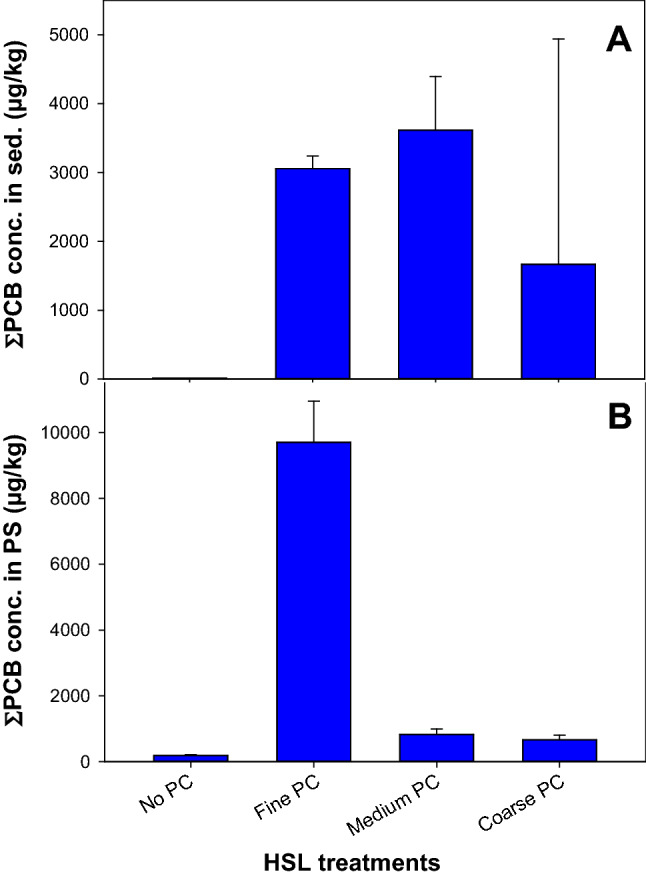
Average (± 1 standard deviation, *n* = 4) concentration of ΣPCBs in bulk sediment (**A**) and in PS (**B**) following ex situ passive sampling for HSL sediment and HSL amended with PCB-containing PC (fine, medium or coarse particle sizes)

**Fig. 2:**
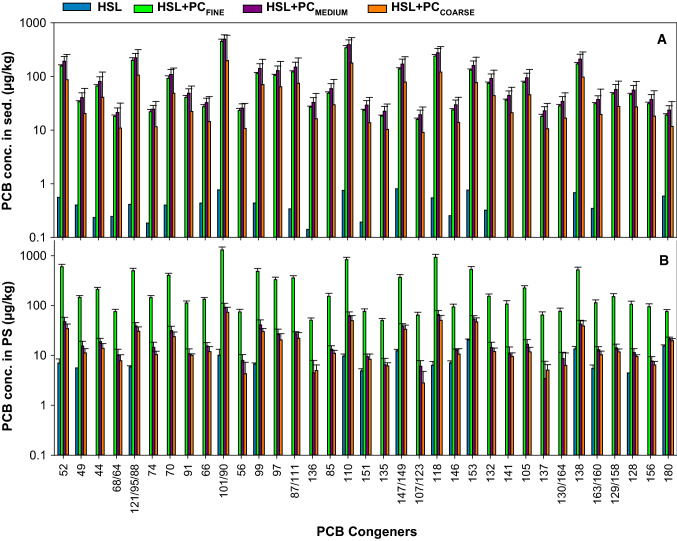
Average (± 1 standard deviation) concentrations of PCB congeners in sediment (**A**) and in PS (**B**) for HSL sediment and HSL amended with fine (HSL + PC_FINE_), medium (HSL + PC_MEDIUM_) or coarse (PCB-containing PCs (HSL + PC_COARSE_). Analytes contributing < 0.5% to the ΣPCBs concentration were omitted

The ∑PCBs concentration (sum of 161 detected congeners) for the HSL + PC treatments (averages = 3.0, 3.6, and 1.7 mg/kg for HSL + PC_FINE_, HSL + PC_MEDUM_, and HSL + PC_COARSE_, respectively) is shown in (Fig. 1). The variability in the concentrations of ∑PCB (Fig. 1) and PCB congeners (Fig. 2) in the PC sediment treatments was relatively low for HSL + PC_FINE_ (coefficient of variation [CV] = 5% for ∑PCB and 4 to 13% for congeners) and HSL + PC_MEDIUM_ (CV = 22% for ∑PCB and 17 to 24% for congeners) but much higher for HSL + PC_COARSE_ (CV = 196% for ∑PCB and 191 to 197% for congeners). The averaged concentration for HSL + PC_COARSE_ was much lower than the target concentration and also lower than for HSL + PC_FINE_ and HSL + PC_MEDUM_. The lower-than-expected measured concentration and the high variability for the coarse PC are explained by the expected high heterogeneity of PC in the sediment caused by the low density of PC for that size fraction. As a result, the concentration of PC for the whole jar was likely higher than the concentration measured in analytical subsamples.

The ∑PCBs concentration for the HSL + PC treatments was substantially higher than concentrations in the HSL sediment prior to addition of PC (0.013 mg/kg) (Fig. 1). Therefore, amending HSL sediment with PC caused an increase in the average concentration of ∑PCB of 231-, 277-, and 131-fold for HSL + PC_FINE_, HSL + PC_MEDIUM_, and HSL + PC_COARSE_, respectively. Similar differences in concentrations occurred for PCB congeners (Fig. 2), and addition of PC promoted increases from 33 to 585-fold (average = 228 ± 149) for HSL + PC_FINE_, from 40 to 644-fold (average = 271 ± 168) for HSL + PC_MEDIUM_, and from 20 to 258-fold (average = 126 ± 74) for HSL + PC_COARSE._ The mass contribution of native PCBs to the total mass of PCBs in the HSL + PC treatments was only approximately 0.5%.

Despite the overall relative similarity in paint-associated PCB mass loading in the sediment for the PC size treatments, the concentrations in the PS depended on the PC size for both ∑PCBs (Fig. 1) and PCB congeners (Fig. 2). Average concentrations in the PS for HSL + PC_FINE_ exceeded that for HSL + PC_MEDIUM_ by 12 times for ∑PCBs and by 4 to 19 times (average = 11) for the dominant congeners. The average concentrations in the PS for HSL + PC_MEDIUM_ exceeded that for HSL + PC_COARSE_ for all but two congeners, and the average exceedance was 1.3-fold. Detailed analytical data are provided in the Supplementary Materials.

The concentrations of PCBs congeners in the sediment and in PS differed widely between the HSL (no PC) and HSL + PC treatments, especially between HSL and HSL + PC_FINE_ (Fig. 2). Therefore, to best illustrate differences in bioavailability relative to bulk sediment concentrations between native PCBs in the HSL, and PCB-containing PC in HSL + PC treatments, we calculated PSAF values. For each PE replicate, PCB concentrations in the PS were divided by the bulk PCB concentration in the sediment normalized by the OC content of the HSL sediment (3.6%).

**Fig. 3:**
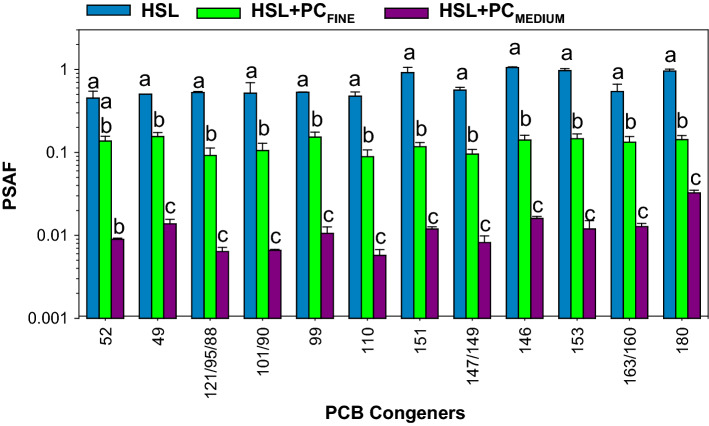
Average (± 1 standard deviation, *n* = 4) PSAF for HSL sediment and HSL amended with fine (HSL + PC_FINE_) or medium (HSL + PC_MEDIUM_) PCB-containing PC. Analytes are the only ones detected in all treatments. Treatments that share the same letters are not significantly different from each other when analyzed using pairwise comparisons

For the HSL sediment (no PC added), PSAF could only be calculated for congeners 52, 49, 121/95/68, 101/90, 99, 110, 151, 147/149, 146, 153, 163/160, and 180 which were detected in the sediment and in the PS (Fig. 3). The PSAF values for HSL sediment were much higher than those determined for the HSL + PC_FINE_ and HSL + PC_MEDIUM_ treatments (Fig. 3). For the above congeners, the across-congeners average PSAF for HSL (0.67 ± 0.23) was 5 times higher than the average for HSL + PC_FINE_ (0.13 ± 0.02) and 62 times higher than the average for HSL + PC_MEDIUM_ (0.012 ± 0.007). For ∑PCBs, the average PSAF for HSL (0.53 ± 0.07) was 5 times higher than the average for HSL + PC_FINE_ (0.11 ± 0.01), 64 times higher than the average for HSL + PC_MEDIUM_ (0.008 ± 0.002) and 36 times higher than the average for HSL + PC_COARSE_ (0.015 ± 0.003) (Supplementary Materials Fig. S3). Statistical analysis showed significant differences between all treatments for ∑PCBs (Supplementary Materials Fig. S3) and for all congeners except for congener 52, the only congener for which HSL was not significantly different from HSL + PC_FINE_ (Fig. 3).

The bioavailability of PC-associated PCB congeners depended on the PC size (Fig. 3 and Supplementary Materials Fig. S4). The average PCB congener PSAF for HSL + PC_FINE_ (0.12 ± 0.03) exceeded that for HSL + PC_MEDIUM_ (0.010 ± 0.005) by 12 times and that for HSL + PC_COARSE_ (0.018 ± 0.009) and by 7 times. The average PCB congener PSAF for HSL + PC_COARSE_ exceeded that for HSL + PC_MEDIUM_ by 1.7 times. Statistical analysis showed significant differences between HSL + PC_MEDIUM_ and HSL + PC_FINE_ but no differences between HSL + PC_MEDIUM_ and HSL + PC_COARSE_ for all congeners (Supplementary Materials Fig. S4).

The higher PSAF for HSL + PC_COARSE_ than for HSL + PC_MEDIUM_ may be attributed to underestimation of sediment concentrations because of the more heterogeneous and variable distribution of PC in the HSL + PC_COARSE_ sediment (Fig. 1) discussed above. Because of this source of uncertainty, HSL + PC_COARSE_ was not compared with HSL in Fig. 3.

The concentration of PCB congeners in the PS remained relatively unchanged (*i.e.*, not statistically different) during increasing mixing periods (Supplementary Materials Fig. S5). The absence of a substantive change in PCB concentrations in the PS with longer mixing times demonstrated that equilibrium (or near-equilibrium) among matrices (*i.e.*, PC, sediment, and PS) was achieved within 60 d of mixing.

### Bioavailability Evaluation Using a Historically Contaminated Sediment Amended with PC

**Fig. 4:**
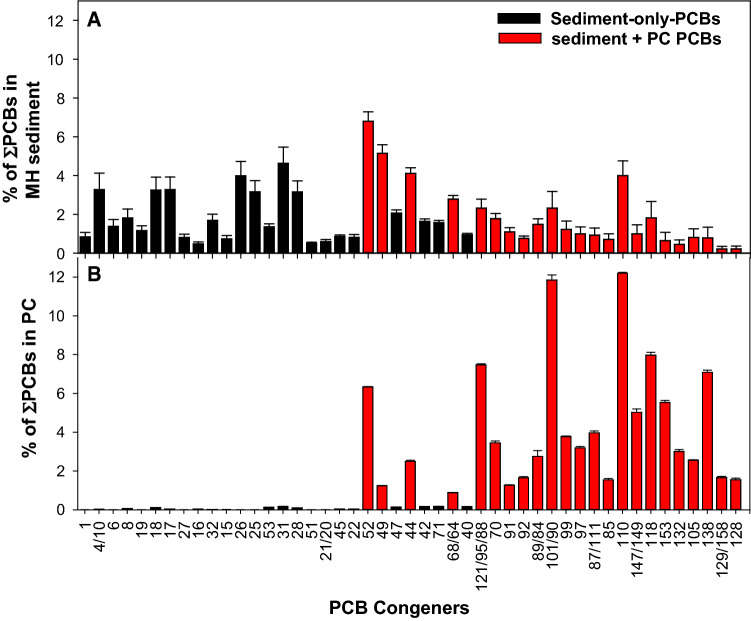
PCB congener profiles for MH sediment (**A**) and for PC (**B**) for select analytes. The average (± 1 standard deviation, *n* = 4) percent contribution of each analyte was derived by dividing its concentration by the ΣPCBs concentration. Congeners that were relatively abundant in the MH sediment but were present only at very low concentrations or non-detects in the PC (referred to as “sediment-only-PCBs”) are shown in black. Analytes present in both MH

The MH sediment had a markedly different homolog group profile compared to PCB-containing PC. The MH sediment had much higher proportional concentrations of mono-, di- and tri-chlorobiphenyls but lower proportional concentrations of penta-, hexa- and hepta-chlorobiphenyls (Supplementary Materials Fig. S6). According to Marti and Armstrong (1990), the combination of PCBs in Manistique indicates dominance by Aroclors 1242 + 1248 for sediment collected at the mouth of the Manistique River. Some of the congeners that were relatively abundant in the MH sediment were present only at very low concentrations or were non-detect in the PC. These congeners will be referred to as “sediment-only-PCBs,” and the most abundant ones are shown in Fig. 4. PCB congeners present in PC and also present in the MH sediment will be referred to as “sediment + PC PCBs” and are also shown in Fig. 4. The concentrations of PCBs congeners in the sediment and in PS are shown in Supplementary Materials Fig. S7, and detailed analytical data are provided in the Supplementary Materials.

For some of the PCBs present in PC (*i*.*e*., 121/95/88, 101/90, 99, 97, 111, 110, 149, 118, 153, 132, 105, 138, 158, and 128), their relative abundance in the MH sediment was much lower than in PCB-containing PC (Supplementary Materials Fig. S8). Therefore, it was expected that for those congeners adding PC to the MH sediment would result in the bulk of the mass present being associated with the PC as opposed to the MH sediment and enable bioavailability comparison between the two sources of PCBs (PC vs MH sediment). This expectation was met for a number of congeners, as shown in Supplementary Materials Fig. S8. For thirteen analytes, the average concentration in the sediment is more than doubled when PC was added to MH sediment (MH + PC_FINE_ treatment), indicating that for those congeners, 50% or more of the bulk PCB mass for that treatment was derived from the PC allowing comparison of their bioavailability with the bioavailability of native congeners measured for the in MH sediment, for which 100% of the mass was sourced from sediment particles.

**Fig. 5:**
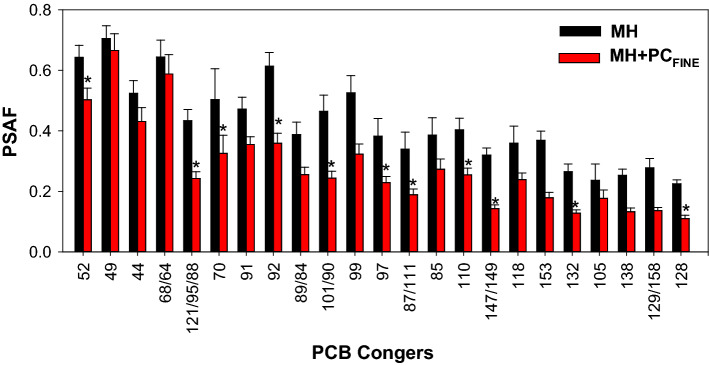
Average (± 1 standard deviation) PSAF following ex situ passive sampling for the MH and MH + PC_FINE_. Analytes are those corresponding to the red bars in Fig. 4. Asterisk (*) indicates significant difference between treatments

Addition of PC to the MH sediment resulted in a 41% increase in the bulk concentration of ΣPCBs in MH + PC_FINE_ relative to MH but resulted in varying increases in congener-specific average concentrations (Supplementary Materials Fig. S9). The percent increases were highest for congeners that were scarce in the sediment but prominent in the PC (Supplementary Materials Fig. S8). The percent increase (from 1 to 303%) in bulk concentration resulting from amending the historically contaminated MH sediment with PC was much higher than the percent increase for the PS (from 0 to 101%). The overall impact of adding PC to the MH sediment is illustrated by comparing the PSAF for MH and MH + PC_FINE_ (Fig. 5). The PSAF was calculated for each replicate by dividing the PCB concentrations in the PE by the bulk PCB concentration in the sediment normalized by the average OC content of the MH sediment (6.7%). For PCB congeners, when the measured bulk sediment concentration increased by over 100% (twofold) from the presence of PC, the PSAF decreased by up to 55% (Fig. 5).

The effect of PC addition on the bioavailable fraction of native PCBs in MH was also evaluated. For congeners that were relatively abundant in the MH sediment but were virtually absent from PC (*i.e.*, sediment-only-PCBs), the PSAF were typically lower for MH + PC_FINE_ by 1 to 4% (but not significantly) than for MH, indicating no meaningful change in bioavailability of native PCBs in the presence of PC (Supplementary Materials Fig. S10).

### Differential Bioavailability

**Fig. 6:**
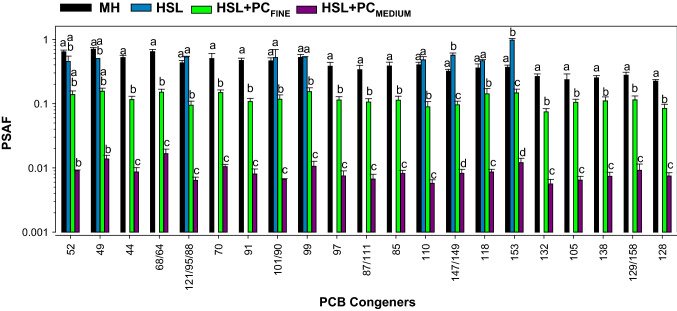
Average PSAF following ex situ passive sampling for HSL, MH and for PCB-containing fine PC amended sediment (HSL + PC_FINE_ and HSL + PC_MEDIUM_) treatments. Analytes are those corresponding to red bars in Fig. 4 with PCBs 84, 89 and 92 omitted because of analytical interference in HSL + PC. Treatments that share the same letters are not significantly different from each other when analyzed using pairwise comparisons

Due to differences in bulk sediment concentrations of the various treatments used in the present study, as well as differences in OC for the HSL and MH sediments in the sediment, bioavailable fraction comparisons among the HSL, MH, MH + PC_FINE_ and HSL + PC_MEDIUM_ treatments are best achieved by comparing PSAFs. For congeners shown in Fig. 6, the average PCB congener PSAF for MH (0.41 ± 0.14) was similar to the average PSAF for HSL (0.56 ± 0.14), but PSAF values for MH were on average 3.5 times higher than those for HSL + PC_FINE_ (0.12 ± 0.02) and 49 times higher than those for HSL + PC_MEDIUM_ (0.009 ± 0.003). Even greater differences were obtained for PSAF values between HSL and HSL + PC_FINE_ (average of fivefold) and HSL and HSL + PCF_MEDIUM_ (average of 65-fold), demonstrating lower bioavailability for PCBs derived from PC dispersed in sediment compared to PCBs sorbed to sediments obtained from field sites and much more so for medium size fraction PC. Statistical analysis showed no significant differences between non-PC treatments (*i.e.*, HSL and MH) for all congeners except for congener 147/149 and 153. The MH treatment was significantly different from HSL + PC_MEDIUM_ for all congeners, but was not significantly different from HSL + PC_FINE_ for congeners 52, 49, and 70 (Fig. 6). Statistical differences among HSL, HSL + PC_FINE_ and HSL + PC_MEDIUM_ are best illustrated in Fig. 3.

## Discussion

To the authors’ knowledge, the present study is the first addressing the bioavailability for PCB-containing PC dispersed in sediment. Bioavailability of PCB from PC in sediment was successfully measured using ex situ polymer sampling, which provides reliable estimates of potential for benthic bioaccumulation. Virtually unchanged PS concentrations of PCBs after 60, 119 and 158 d confirmed effective equilibrium conditions between the PC + sediment matrix and PS at 60 d indicating that desorption of PCBs from PC approached a plateau after 60 d of mixing with sediment.

PC size had a strong effect on the bioavailable fraction after 60 d of sediment and PC mixing, as HSL + PC_FINE_ generated ΣPCBs equilibrium average concentration in the PS 12‐fold higher than for HSL + PC_MEDIUM_ and 15‐fold higher than for HSL + PC_COARSE_. Therefore, particles in the range of 0.25 to 5 mm resulted in over one order of magnitude less accumulation in the PS compared to the < 0.045 mm fraction. The surface‐area‐to‐volume ratio for the fine PC was approximately 6 times higher than for the medium PC and approximately 5 times higher relative to the coarse particles. Therefore, desorption of PCBs from PC increases with increasing surface‐area‐to‐volume ratio. The average concentration in the PS for the coarse fraction was similar to that for the medium fraction as expected based on the similar surface‐area‐to‐volume ratio for those size fractions. Although the relation between desorption or bioavailability and particle size has not been previously evaluated for organic contaminants associated with PC, such a trend has been reported for copper associated with PC (Turner et al. 2008). Desorption of PCB associated with fragment-type microplastics also decreased with increasing surface‐area‐to‐volume ratio (Endo et al. 2013; Lee et al. 2018). The relation between surface‐area‐to‐volume ratio and bioavailability is likely explained by faster desorption rate for surface PCBs than subsurface PCBs in PC.

Because the accumulation in the PS did not increase with longer mixing times, nearly all of the PCBs mass desorption from the fine size paint particles was assumed to have occurred within 60 d. This observation is consistent with the substantial decrease in the rate of release of PCBs from PC in water after day 21 of the leaching study (Uhler et al. in 2021). Because the leaching study used PC mechanically reduced to a size that would pass through a 9.5 mm standard sieve, desorption of PCBs from medium and coarse PC used in this study (0.3 to 5 mm in diameter) was expected to be fairly slow after 60 d. Therefore, larger size fractions would not be expected to reach the bioavailability of the fine particle sizes if mixed for longer periods.

No published data on the particle‐size distribution specifically for PCB‐containing paint particles in sediment were found during this research. However particle size data have been generated for antifouling paint particles sampled from harbors and vessel maintenance sites (USEPA 1979; Muller‐Karanassos et al. 2019; Wu et al. 2016). Based on this literature information, spent paint particles in sediments originating from harbors and vessel maintenance sites appear to primarily occur in > 0.2‐mm size class which correspond to the medium size PC used in the present study.

Investigation of differential bioavailability is essential for understanding environmental fate and for evaluation environmental risks associated with the contaminated anthropogenic matrices present in sediments (Beckingham and Ghosh 2017). The differential bioavailability between HSL (all PCB mass associated with sediment particles) and HSL + PC treatments (~ 99.5% of PCB mass associated with PC) is illustrated in Fig. 3. For the congeners shown in Fig. 3, the PSAF for HSL exceeded that for HSL + PC_FINE_ on average by 5 times and that for HSL + PC_MEDIUM_ by 62 times. Differential bioavailability was also evaluated using sediment from MH with a much higher mass of PCBs associated with sediment particles compared to HSL sediment (Fig. 6). Despite orders on magnitude differences in sediment PCBs concentration, the PSAF for MH were similar than those for HSL. The PSAF for those sediments represent the bioavailable fraction for PCBs associated with sediments as an additional point of comparison for sediment-amended paint-chips-associated PCBs (Fig. 6). The PSAF for MH was 3.5 times higher that than for HSL + PC_FINE_ and 47 times than that for HSL + PC_MEDIUM_ for the sum of the most abundant congeners in PC. Furthermore, addition of fine PC to MH sediment caused a 30% overall decrease in PSAF for sediment + PC PCBs (those present both in MH and in the PC) as indicated by comparing the averages for MH + PC_FINE_ and MH (Fig. 5).

The results from the present study indicate that PCBs are strongly sorbed to PC. In a related study of the leaching of PCBs from PC, Uhler et al. (2021) reported that more than 99% of the PCBs in the PCB-paint chips remained trapped in the paint matrix at the cessation of leaching after 1,150 days (*i.e.*, less than 1% of the PCB mass desorbed into the water). This finding is highly indicative of much stronger sorption of PCBs to PC than to sediment, as over 50% of the PCBs desorbed from suspended sediments from contaminated sites in the USA (*i.e.*, Hunters Point Naval Shipyard, Lake Hartwell and Hudson River) over periods ranging from days to weeks (Carroll et al. 1994; Zimmerman et al. 2004; Werner et al. 2005). A proposed mechanism that explains the observed partitioning behavior of PCBs from PCB PC is a process where surface bound PCB molecules partition relatively rapidly into water in contact with the paint chips; the rate of desorption decreases as the PCB molecules on the paint surface become depleted. Ultimately, the majority of the PCBs have slow matrix diffusion out of the PC and into the surrounding water. According to this proposed mechanism, PCBs partitioning from PC into overlying or porewater are thus inherently limited to a very small fraction of the total PC PCBs mass that is located at the surface of the PC. During the PC and sediment mixing in the present study (for up to 159 days), PCBs were desorbing from pre-loaded PC into the surrounding pore water and resorbing to the clean sediment matrix. Low desorption of PCBs from PC (especially for particles in the range of 0.25–5 mm), relative to desorption from the sediment matrix, would explain the results obtained in the present study. Low desorption of DDT and its major transformation products from PC as a result of strong sorption for those compounds to the paint matrix has also been shown, as less than 10% of the total mass of those compounds desorbed to water within 7 d under active mixing (Wu et al 2016). Desorption rates are expected to be lower under low mixing in situ conditions.

In the present experiment, the mass of PC added to MH sediments corresponded to only to 0.011–0.012% the mass of dry sediment. Even though PC likely acted as a strong sorbent for sediment PCBs, their presence at such low dosing level failed to result in a significant effect on the bioavailability of native PCBs in the MH sediment (Supplementary Materials Fig. S10). Much higher doses of highly sorptive materials have been shown to decrease bioavailability. For example, decrease in PCB bioavailability by microplastics was associated with a polypropylene dose of 5% by dry wt. (Beckingham and Ghosh 2017).

The differential bioavailability results generated in the present study demonstrate the much lower bioavailability for PC-PCBs relative to native PCBs historically associated with sediment particles. This is especially relevant considering that regulators are increasingly recognizing the value of bioavailability-based assessment of contaminated sites for management decisions (Greenberg et al. 2014; Booij et al. 2016). Information from this study on the bioavailability of PCBs from PC will allow environmental scientists to assess the potential environmental effects of PCB-containing paint chips in areas where their release to the environment has occurred. Differential bioavailability information is expected to be particularly relevant for use in risk assessment and remediation decision-making for areas where sediment contaminated with PCB originating from sources other than PC is co-located with PCB PC, such as the Swan Island Lagoon, located in the Portland Harbor (OR, USA) (Oregon Department of Environmental Quality 2020) and many other sites (Supplementary Materials). To our knowledge, this is the first study investigating differential bioavailability for paint or other manufactured product containing hydrophobic organic chemicals added as an ingredient during manufacturing. The research framework provided in the present study is also applicable for investigating the differential bioavailability of contaminants associated with other manufactured materials which have been found in sediment and soil such as DDT-containing paint (Lin et al. 2009; Wu et al. 2016) and PCB-containing materials such as plaster and caulk (Andersson et al. 2004; Ruus et al. 2006; Herrick et al. 2007; Davies and Delistraty 2016).

## Conclusions

PCB were used as performance additives to paint formulations between the 1940s and early 1970s. Despite reports of sediment and soil contamination by PCB-containing PC at multiple field sites, no known previous scientific study evaluating the bioavailability potential of PCB-containing PC in aquatic environments exists. This study comparatively evaluated the bioavailability potential of PCB-containing PC dispersed in sediments with no known PCB impact (HSL sediments) and in with sediments with historical PCB impacts from non-paint sources (MH sediments). PC size had a strong effect on the bioavailable PCB fraction, as fine particles (< 0.045 mm) generated ∑PCBs concentration in the PS over one order of magnitude higher than for coarser particles (0.25—5 mm), which has been shown to be the prevalent PC fraction dispersed into environment as a result of vessel maintenance activities. Differential bioavailability was successfully assessed using PSAF, a novel approach that is analogous to the biota-sediment-accumulation factor (BSAF), but simpler to apply because of the many complexities associated measuring bioaccumulation. The PSAF for the historically contaminated MH sediments were ~ 50–60 times higher than those for the 0.25–5 mm PC mixed in non-PCB impacted HSL sediments. This indicates much lower bioavailability for PCBs associated with PC compared to bioavailability from sediment historically contaminated with PCBs from non-paint sources. Forensic investigations have made it clear that sites have multiple sources of PCBs (including and excluding PC). This study shows that bioavailability of PCBs from sources such as PC present in sediment is not directly proportional to the bulk phase concentrations.

## Supplementary Information

Below is the link to the electronic supplementary material.

Supplementary file1 (DOCX 676 KB)

Supplementary file2 (XLSX 256 KB)

## Data Availability

Data are available from the corresponding author (guilherme.lotufo@usace.army.mil).
