# Supplementary material for: Passive-Sampler-Based Bioavailability Assessment of PCB Congeners Associated with Aroclor-Containing Paint Chips in the Presence of Sediment
Source: Arch Environ Contam Toxicol. 2021 Dec 17;82(1):105–18. doi: 10.1007/s00244-021-00907-2 (PMC8732844; doi:10.1007/s00244-021-00907-2)
Supplement: Supplementary file 1 — Supplementary file1 (DOCX 676 KB) [file 244_2021_907_MOESM1_ESM.docx]

**Passive-sampler-based bioavailability assessment of PCB congeners associated with Aroclor-containing paint chips in the presence of sediment**

Guilherme R. Lotufo^1^, Philip T. Gidley^1^, Andrew D. McQueen^1^, David W. Moore^1^, Deborah A. Edwards^2^, Jeffery Hardenstine^3^, Allen D. Uhler^3^

^1^ US Army Engineer Research and Development Center, Vicksburg MS

^2^ ExxonMobil Environmental and Property Solutions Company, Spring, Texas 77389

^3^ NewFields-Environmental Forensics Practice, Rockland MA

**SUPLEMENTARY MATERIALS**

**Historic use of** **PCB-containing paint**

PCBs have been used until 1970s in several nonelectrical applications as additives to a variety of materials including paints (Scott and Snyder 2015; Fu et al. 2003; Hess et al. 2001). PCBs were added to paint to give the paint better adhesive properties and to provide anti-corrosion protection from moisture, chemicals and flames (Jensen et al. 1972; Johnsen and Engøy 2000; Martin and Richards 2010). Aroclor 1254 was the most common plasticizer added to paints during the 1950s, 1960s, and early 1970s (Scott and Snyder 2015). Chemical-resistant chlorinated rubber paints contained various Aroclors typically at 10-12% of the total composition (Scott and Snyder 2015). PCBs in paint from a former nuclear test reactor in Savannah, USA, were present at concentrations as high as 6% (Lowry et al. 1998). Concentrations of PCBs as high as 7.4% were found in samples of paint sampled during a remediation project from a former military base in Arctic Canada (Poland et al. 2001).

Paints containing PCBs were commonly used as vessel coating from the 1940’s (Martin and Richards 2010) until approximately the mid-1980s, when ship maintenance companies consumed post-PCB ban stocks of PCB-containing paints. Studies of PCBs in World War II era US Navy ships that have been scrapped have shown that PCBs were present in a variety of materials including paint, where PCBs were used as plasticizing agents (Shields and Pietari 2010; Shields et al. 2017). PCB paint as commonly used to coat the surfaces of many post-World War II ships and submarines, including those used by the navy in the USA and other countries (Larcomb et al. 1996; Hess et al. 2001; Still et al. 2003; USEPA 2006, 2013; George et al. 2006; Johnston et al. 2006; Martin and Richards 2010). PCB-containing paints were also used in industrial, military and in urban buildings including schools (Poland et al. 2001; Ruus et al. 2006; Jartun et al. 2009a; US Army Corps of Engineers 2012; Saitta et al. 2015; Brown et al. 2016). Chlorinated paraffin replaced PCB-paints, as the post-production stocks of PCB-paint were consumed (Martin and Richards 2010).

**Environmental contamination by PCB-containing paint**

Renovations, repairs, and weathering have caused paints containing PCBs to flake, which may have resulted in the contamination of water, sediment, and soil (Saitta et al. 2014). According to Battelle (2012), paints containing PCBs should be considered a potential source of in forensic investigations of contaminated sediment sites. Numerous studies have identified vessel maintenance activities, including power washing of vessels and removal of old paint (including paints containing PCBs) via sand blasting have been identified as a potential source of PCB contamination in aquatic environments (Jensen et al. 1972; Johnsen and Engøy 2000; Gold and Bloom 2000; Still et al. 2003; Martin and Richards 2010; Bellucci et al. 2016). In addition, ship recycling operations may generate a large mass of PCB containing paint, which may contaminate the surrounding environment (Andersen et al. 2000; Hess et al. 2001).

When paint containing PCBs flakes is released during natural weathering or is sand blasted off of land structures in urban and industrial areas, the dust has been shown to deposit on the ground surface becoming available to move into surface soil, surface water and sediment through infiltration and runoff processes. Therefore, flaking PCB paint from land structures are considered a relevant source of PCB contamination to aquatic and terrestrial environments (Andersson et al. 2004; Kuusisto et al. 2007; Jartun et al. 2008, 2010; Wischkaemper et al. 2013; King County 2013; Davies and Delistraty 2016). Paints containing PCBs remaining in sunken ships after removal efforts have also been identified as a potential source of environmental contamination (George et al. 2006; Johnston et al. 2006; USEPA 2006; Čulin and Bielić 2015).

Paints containing PCBs were reported as a relevant source of contamination at numerous aquatic and terrestrial sites, as detailed below.

- Bioaccumulation of PCBs in zooplankton has been attributed to their release from vessel paint (Jensen et al. 1972). Concentrations up to 170 mg/kg-lipids (as ∑PCB, congeners not specified) were reported.
- The PCB-profiles (relative concentrations of the congeners) of blue mussels were similar to the PCB-profile of the paint and plaster, thus it was concluded residue PCB-containing plaster and paint originating from the façade of a building located on the shore of a fjord in Norway and was believed to be the main source of high PCB body residues in blue mussels (Russ and Green 2002; Russ et al. 2006). The PCB-concentrations in blue mussels sampled near the building was variable over the years, and the highest measured concentration was 1.1 mg/kg-wet-wt (∑PCB_7_ , i.e., sum concentration of congeners PCB-28, -52, -101, -118, -138, -153, -180 ) (Ruus and Green 2002). It was also speculated that local cod may have ingested paint and plaster particles present in the water column (Russ et al. 2006).
- Release of paint residues from ship repair yards and direct release from the hull during port calls were responsible for a considerable fraction of contaminants, including PCBs, associated with sediments outside of ship repair yards and around quay structures in Norway (Johnsen and Engøy 2000). The mean concentration of PCBs in sediment samples was 0.55 mg/kg (as ∑PCB_7_).
- Routine boatyard activities, including power washing of boats, as well as natural sloughing off of the paints from the bottom of the boats were considered the main source of PCBs contamination in sediment and soil in the vicinity of a former boat yard in Apponagansett Bay, South Dartmouth, MA (Martin and Richards 2010). The concentration of PCBs in sediment samples were below 1 mg/kg (as ∑PCB, congeners not specified). Concentrations of ∑PCB in soils were as high as 10 mg/kg.
- The Swan Island Lagoon, located in the Portland Harbor (OR, USA) along the eastern bank of the Willamette River, is an area where elevated concentrations of PCBs and other organic and trace metal contaminants have been documented (Lower Willamette Group 2016). The north, south and west perimeters of Swan Island Lagoon has been the site of commercial ship repair and maintenance activities since 1942 (Oregon Department of Environmental Quality 2020). Generally co-located, elevated concentrations of PCBs, heavy metals and tributyltin in sediments proximal to Swan Island dry dock and berthing operations suggest that the contaminants, including PCBs, are associated with paint chips and sandblasting solids associated with the ship maintenance and recoating that were released to the environment. The concentration of PCBs in sediment reported for the site was 0.71 mg/kg (as ∑PCB, congeners not specified) (Oregon Department of Environmental Quality 2020).
- Concentrations as high as 1.3 mg/kg (as the sum of 32 congeners) were measured in sediment at sites potentially contaminated with PCB-containing paint due to their proximity to dry docks in Italy where PCB paints were used (Belluci et al. 2016).
- Contamination of sediment in the vicinity of Todd Shipyard in Tacoma, Washington was at least in part a result of release of PCBs from various sources during and after manufacturing activities, including paint containing PCBs (Shields et al. 2017). The concentration of PCBs in site sediment was not reported.
- Building materials, including paint containing PCBs, transported via stormwater runoff, were the suspected source of sediment contamination in the harbor area of Bergen, Norway (Anderson et al. 2004). The concentrations of PCBs in sediment were not reported.
- PCBs present in paint used in wooden and concrete buildings along a seaside urban area in Norway dispersed into the aquatic environment and was believed to have caused significant sediment contamination (Jartun et al. 2008, 2009a). Concentrations in the urban runoff sediments were as high as 0.7 mg/kg.
- Concentrations as high as 0.83 mg/kg (as t∑PCB_7_) were present in sediment sampled from the harbor area of Trondheim, in Norway. The main source of contamination was proven to be old exterior paint chips reaching the harbor via urban runoff (Jartun and Pettersen 2010).
- High levels of PCBs in paint, caulk, and other building materials have been found in areas near Lower Duwamish Waterway in Seattle (WA, USA) and were considered potential source of sediment contamination (Davies and Delistraty 2016). The concentrations of PCBs at sites potentially contaminated with PCBs originating from building materials were not reported.
- Concentrations as high as 1.4 mg/kg were reported for soils contaminated by paints containing PCBs released during ship decommissioning activities in Bangladesh has been reported (Andersen et al. 2000).
- Flaking of paint containing PCBs from building refuse, scrap metal and other materials were considered the main source of contamination of soils form Spitsbergen Island, Norway (Jartun et al. 2009b). Concentrations as high as 29 mg/kg (as t∑PCB_7_) were reported at contaminated soil sites.

**References**

Andersen, A., Bjornbom, E., & Sverud, T., 2000. Decommissioning of ships - Environmental standards. Shipbreaking practices/onsite assessment Bangladesh, Chittagong. *Oslo, Norway: Det Norske Veritas*. https://shipbreakingplatform.org/wp-content/uploads/2018/11/dnvbangladesh.pdf

Andersson, M., Ottesen, R.T. and Volden, T., 2004. Building materials as a source of PCB pollution in Bergen, Norway. Science of the Total Environment, 325(1-3), pp.139-144. https://doi.org/10.1016/j.scitotenv.2003.11.014

Battelle, 2012 A handbook for determining the sources of PCB contamination in sediments. NAVFAC Engineering and Expeditionary Warfare Center.TR-NAVFAC EXWC-EV-1302, 164 pp. https://clu-in.org/download/contaminantfocus/pcb/pcb_sediment_handbook.pdf.

Bellucci, L.G., Cassin, D., Giuliani, S., Botter, M. and Zonta, R., 2016. Sediment pollution and dynamic in the Mar Piccolo of Taranto (southern Italy): insights from bottom sediment traps and surficial sediments. Environmental Science and Pollution Research, 23(13), pp.12554-12565. https://doi.org/10.1007/s11356-016-6738-6

Brown, K.W., Minegishi, T., Cummiskey, C.C., Fragala, M.A., Hartman, R. and MacIntosh, D.L., 2016. PCB remediation in schools: a review. Environmental Science and Pollution Research, 23(3), pp.1986-1997. https://doi.org/10.1007/s11356-015-4689-y

Čulin, J. and Bielić, T., 2015. Ship-source pollution by polychlorinated biphenyls and brominated flame retardants. *Pomorstvo*, *29*(1), pp.90-94. https://hrcak.srce.hr/file/207017

Davies, H. and Delistraty, D., 2016. Evaluation of PCB sources and releases for identifying priorities to reduce PCBs in Washington state (USA). Environmental Science and Pollution Research, *23*(3), pp.2033-2041. https://doi.org/10.1007/s11356-015-4828-5

Fu, J., Mai, B., Sheng, G., Zhang, G., Wang, X., Xiao, X., Ran, R., Cheng, F., Peng, X., Wang, Z. and Tang, U.W., 2003. Persistent organic pollutants in environment of the Pearl River Delta, China: an overview. Chemosphere, *52*(9), pp.1411-1422. https://doi.org/10.1016/S0045-6535(03)00477-6

George, R.D., In, C.R., Johnston, R.K., Seligman, P.F., Gauthier, R.D. and Wild, W.J., 2005. Seawater leaching investigation of polychlorinated biphenyls from solid matrices. In OCEANS, 2005. Proceedings of MTS/IEEE (pp. 1492-1500). IEEE, Washington, D.C. . https://doi.org/10.1109/OCEANS.2005.1639967

Gold, S.B. and Bloom, D.M., 2000. Painting the PCB picture. Environmental Protection, 11(10), p. 58. https://eponline.com/articles/2000/10/01/painting-the-pcb-picture.aspx?m=1

Hess, R.W., Rushworth, D., Hynes, M.V. and Peters, J.E., 2001. Disposal options for ships. RAND National Security Research Division. Santa Monica, CA. https://doi.org/10.7249/MR1377

Jartun, M., Ottesen, R.T., Steinnes, E. and Volden, T., 2008. Runoff of particle bound pollutants from urban impervious surfaces studied by analysis of sediments from stormwater traps. Science of the Total Environment, *396*(2-3), pp.147-163. https://doi.org/10.1016/j.scitotenv.2008.02.002

Jartun, M., Ottesen, R.T., Steinnes, E. and Volden, T., 2009a. Painted surfaces–Important sources of polychlorinated biphenyls (PCBs) contamination to the urban and marine environment. Environmental Pollution, *157*(1), pp.295-302. https://doi.org/10.1016/j.envpol.2008.06.036.

Jartun, M., Ottesen, R.T., Volden, T. and Lundkvist, Q., 2009b. Local sources of polychlorinated biphenyls (PCB) in Russian and Norwegian settlements on Spitsbergen Island, Norway. Journal of Toxicology and Environmental Health, Part A, 72(3-4), pp.284-294. https://doi.org/10.1080/15287390802539426

Jartun, M. and Pettersen, A., 2010. Contaminants in urban runoff to Norwegian fjords. Journal of Soils and Sediments, 10(2), pp.155-161. https://doi.org/10.1007/s11368-009-0181-y

Jensen, S., Renberg, L. and Olsson, M., 1972. PCB contamination from boat bottom paint and levels of PCB in plankton outside a polluted area. Nature, 240, pp. 358-360. https://doi.org/10.1038/240358b0

Johnsen A, Engøy T., 2000. Contamination from Marine Paints—A Norwegian Perspective. Defense Technical Information Center Compilation Part Notice ADPO10602. Part of report: Approaches to the implementation of Environment Pollution Prevention Technologies at Military Bases

Johnston, R.K., George, R.D., Richter, K.E., Wang, P.F. and Wild, W.J., 2006. Ex-ORISKANY Artificial Reef Project, Prospective Risk Assessment Model (PRAM) Version 1.4c”. Prepared for Naval Sea Systems Command, Washington, D.C. https://apps.dtic.mil/sti/pdfs/ADA485008.pdf

King County, 2013. PCB/PBDE loading estimates for the Greater Lake Washington Watershed. Prepared by Curtis DeGasperi, Water and Land Resources Division. Seattle, Washington. https://your.kingcounty.gov/dnrp/library/water-and-land/watersheds/cedar-river-lake-wa/lake-washington-pcb-pbde-loadings/epa-data-report-final-sept2013.pdf

Kuusisto, S., Lindroos, O., Rantio, T., Priha, E. and Tuhkanen, T., 2007. PCB contaminated dust on indoor surfaces–Health risks and acceptable surface concentrations in residential and occupational settings. Chemosphere, 67(6), pp.1194-1201. https://doi.org/10.1016/j.chemosphere.2006.10.060

Larcomb, B.J., Cline, J.M., Merrill, E.A., Jederberg, W.W. and Still, K.R., 1996. Risk assessment of polychlorinated biphenyls (PCBs) on-board Navy ships. NMRI‐96‐72. Operational Technologies, Dayton, OH, USA. https://apps.dtic.mil/sti/pdfs/ADA325398.pdf

Lower Willamette Group, 2016. Final Remedial Investigation (Lower Willamette River, Portland Harbor Superfund Site, U.S. EPA Docket No: CERCLA-10-2001-0240). Submitted to U.S. Environmental Protection Agency, February 8, 2016. https://semspub.epa.gov/work/10/1464370.pdf

Lowry, N.J., Kubilius, W.P., Bell, C.E., Kahal, E.J., Young, J.E., Crump, S.L., Summer, M.E., Fliermans, C.B. and Weber, J., 1998. Analytical Study of High Concentration PCB Paint at the Heavy Water Components Test Reactor (HWCTR) – Report. Westinghouse Savannah River Company. Report No. WSRC-TR-98-00374. https://doi.org/10.2172/4931

Martin, M.E. and Richards, M.J., 2010. PCB and heavy metal soil remediation, former boat yard, South Dartmouth, Massachusetts. Proceedings of the Annual International Conference on Soils, Sediments, Water and Energy, Worcester, MA. https://scholarworks.umass.edu/soilsproceedings/vol14/iss1/19

Oregon Department of Environmental Quality, 2020. Environmental Cleanup Site Information (ECSI) Database Site Summary Report - Details for Site ID 271, Swan Island Upland Facility/Vigor Industrial. https://www.deq.state.or.us/lq/ECSI/ecsidetail.asp?seqnbr=271

Poland, J.S., Mitchell, S. and Rutter, A., 2001. Remediation of former military bases in the Canadian Arctic. Cold Regions Science and Technology,32(2-3), pp.93-105. https://doi.org/10.1016/S0165-232X(00)00022-7

Ruus, A. and Green, N.W., 2002. Measure oriented environmental monitoring of the Sørfjord and Hardangerfjord 2002. Report component 2, Contaminants in organisms. Norwegian State Pollution Monitoring Programme Report no. 865/02. TA-No. 1922/2002 (in Norwegian).

Ruus, A., Green, N.W., Maage, A. and Skei, J., 2006. PCB-containing paint and plaster caused extreme PCB-concentrations in biota from the Sørfjord (Western Norway)—A case study. *Marine pollution bulletin*, *52*(1), pp.100-103. https://doi.org/10.1016/j.marpolbul.2005.11.010

Saitta, E.K., Gittings, M.J., Clausen, C., Quinn, J. and Yestrebsky, C.L., 2014. Laboratory evaluation of a prospective remediation method for PCB-contaminated paint. Journal of Environmental Health Science and Engineering*,* 12(1), pp.1-5. https://doi.org/10.1186/2052-336X-12-57

Saitta, E.K., Gittings, M.J., Novaes-Card, S., Quinn, J., Clausen, C., O'Hara, S. and Yestrebsky, C.L., 2015. Case study of a non-destructive treatment method for the remediation of military structures containing polychlorinated biphenyl contaminated paint. Journal of environmental management, 158, pp.40-47. https://doi.org/10.1016/j.jenvman.2015.04.038

Scott, M. and Snyder, R., 2015. PCBs in construction materials: old chemical, new context. Environmental Claims Journal, 27(3), pp.244-263. https://doi.org/10.1080/10406026.2015.1062660

Shields, W.J., Pietari, J. and Sparacio, T., 2017. Use of PCBs at World War II manufacturing sites. 37th International Symposium on Halogenated Persistent Organic Pollutants (POPs) - DIOXIN 2017. Vancouver, Canada. August 20-25, 2017. http://dioxin20xx.org/wp-content/uploads/pdfs/2017/10022.pdf

Shields, W.J. and Pietari, J., 2010. Historic Reconstruction of Contaminant Releases at Military Shipyards during World War II. Presented at the 2010 Society of Environmental Toxicology and Chemistry Annual Meeting in Portland, Oregon. Abstract available at https://www.setac.org/store/viewproduct.aspx?id=1247052. Additional information provided at https://www.exponent.com/experience/pcb-release-from-wwii-shipyard/?pageSize=NaN&pageNum=0&loadAllByPageSize=true

Still, K.R., Arfsten, D.P., Jederberg, W.W., Kane, L.V. and Larcom, B.J., 2003. Estimation of the health risks associated with polychlorinated biphenyl (PCB) concentrations found onboard older US Navy vessels. Applied Occupational and Environmental Hygiene,18(10), pp.737-758. https://doi.org/10.1080/10473220301444

US Army Corps of Engineers. 2012. PCBs in caulk and paint. Public Works Technical Bulletin 200-1-126. Washington, DC. https://www.wbdg.org/FFC/ARMYCOE/PWTB/pwtb_200_1_126.pdf

Environmental Protection Agency (USEPA), 2006. National Guidance: Best Management Practices for Preparing Vessels Intended to Create Artificial Reefs Polychlorinated Biphenyls (PCBs). National Guidance: Best Management Practices for Preparing Vessels Intended to Create Artificial Reefs (EPA 842-B-06-002) May 2006. https://www.epa.gov/sites/production/files/2015-09/documents/artificialreefguidance.pdf

Environmental Protection Agency (USEPA), 2013. Technical Guidance for Determining the Presence of Polychlorinated Biphenyls (PCBs) at Regulated Concentrations on Vessels (Ships) to be Reflagged. Office of Pollution Prevention and Toxics (OPPT) / National Program Chemicals Division and Office of Resource Conservation and Recovery (ORCR) / Materials Recovery and Waste Management Division http://www3.epa.gov/epawaste/hazard/tsd/pcbs/pdf/pcb_shps_guidnce_fnl.pdf

Wischkaemper, H.K., Beliveau, A.F. and Henderson, R.W., 2013. U.S. EPA region 4 technical services section issue paper for polychlorinated biphenyl characterization at region 4 superfund and RCRA sites. https://www.epa.gov/risk/region-4-issue-paper-pcb-characterization 15 pp. Accessed 5 5 2021

**SUPPLEMENTARY TABLES**

**Table S1. Experimental treatments used in the bioavailability assessment of PCB-containing paint in the presence of sediment.**

| **Treatment** | **Description** | **Objective** |
| --- | --- | --- |
| HSL | Horseshoe Lake sediment obtained from pristine oxbow lake containing relatively low concentrations of PCBs (ΣPCBs concentration = 0.13 mg/kg) | Contaminated sediment for comparison with HSL + PC treatments |
| HSL = PC_FINE_  (60 d mixing) | HSL sediment mixed with fine PC for 60 d targeting a ΣPCBs concentration of 4 mg/kg as PC | Highest surface area PC, assumed as worst-case |
| HSL = PC_FINE_  (119 d mixing) | HSL sediment mixed with fine PC for 119 d targeting a ΣPCBs concentration of 4 mg/kg as PC | Evaluate polymer/sediment-PC time to equilibrium |
| HSL = PC_FINE_  (158 d mixing) | HSL sediment mixed with fine PC for 158 d targeting a ΣPCBs concentration of 4 mg/kg as PC |  |
| HSL = PC_MEDIUM_  (60 d mixing) | HSL sediment mixed with medium PC for 60 d targeting a ΣPCBs concentration of 4 mg/kg as PC | Comparison with fine-PC |
| HSL = PC_COARSE_  (60 d mixing) | HSL sediment mixed with coarse PC for 60 d targeting a ΣPCBs concentration of 4 mg/kg as PC |  |
| MH | Manistique Harbor sediment with PCBs with no known sources of paint chips (ΣPCBs concentration = 5.8 mg/kg) | Contaminated sediment for comparison with HSL + PC treatments |
| MH + PC_FINE_ | MNQ sediment mixed with fine paint chips for 60 d targeting a ΣPCBs concentration of 9.8 mg/kg (4 mg/kg as PC) | Comparison with MH sediment |

**Table S2. Concentration of PAHs, chlorinated pesticides and metals in Horseshoe Lake sediment. RL = reporting limit.**

| **PAHS** | µg/kg | RL | **PESTICIDES** | µg/kg | RL | µg/kg | RL |
| --- | --- | --- | --- | --- | --- | --- | --- |
| Naphthalene | 22.20 | 1.76 | 4,4´-DDD | ND | 0.40 | 0.79 | 0.12 |
| Phenanthrene | 26.20 | 1.76 | 4,4´-DDT | ND | 0.40 | ND | 0.12 |
| Anthracene | 4.70 | 1.76 | 4,4´-DDE | ND | 0.40 | 2.59 | 0.12 |
| Fluoranthene | 48.80 | 1.76 | Aldrin | ND | 0.40 | ND | 0.12 |
| Pyrene | 895.00 | 1.76 | alpha-BHC | ND | 0.40 | ND | 0.12 |
| 2-Methylnaphthalene | 30.60 | 1.76 | alpha-Chlordane | ND | 0.40 | ND | 0.12 |
| Benzo (a) anthracene | 14.30 | 1.76 | beta-BHC | ND | 0.40 | ND | 0.12 |
| Chrysene | 25.30 | 1.76 | delta-BHC | ND | 0.40 | ND | 0.12 |
| Benzo (b) fluoranthene | 24.40 | 1.76 | Dieldrin | ND | 0.40 | ND | 0.12 |
| Benzo (k) fluoranthene | 26.60 | 1.76 | Endosulfan I | ND | 0.40 | ND | 0.12 |
| Benzo (a) pyrene | 17.40 | 1.76 | Endosulfan II | ND | 0.40 | ND | 0.12 |
| 1-Methylnaphthalene | 15.20 | 1.76 | Endosulfan sulfate | ND | 0.40 | ND | 0.12 |
| Indeno (1,2,3-cd) pyrene | 12.30 | 1.76 | Endrin | ND | 0.40 | ND | 0.12 |
| Dibenz (a,h) anthracene | ND | 1.76 | Endrin aldehyde | ND | 0.40 | ND | 0.12 |
| Benzo (g,h,i) perylene | 14.20 | 1.76 | Endrin ketone | ND | 0.40 | ND | 0.12 |
| Acenaphthylene | ND | 1.76 | gamma-BHC (Lindane) | ND | 0.40 | ND | 0.12 |
| Acenaphthene | ND | 1.76 | gamma-Chlordane | ND | 0.40 | ND | 0.12 |
| Fluorene | 4.94 | 1.76 | Heptachlor | ND | 0.40 | ND | 0.12 |
|  |  |  | Heptachlor epoxide | ND | 0.40 | ND | 0.12 |
|  |  |  | Methoxychlor | ND | 0.40 | ND | 0.12 |
|  |  |  | Toxaphene | ND | 16.1 | ND | 4.41 |
| **METALS** | mg/kg | RL |  |  |  |  |  |
| Antimony | 0.503 | 0.0498 |  |  |  |  |  |
| Arsenic | 3.27 | 0.0482 |  |  |  |  |  |
| Barium- | 198 | 0.0482 |  |  |  |  |  |
| Beryllium | 0.73 | 0.0482 |  |  |  |  |  |
| Cadmium | 0.577 | 0.0482 |  |  |  |  |  |
| Chromium | 10.8 | 0.0482 |  |  |  |  |  |
| Cobalt | 7.41 | 0.0482 |  |  |  |  |  |
| Copper | 17.2 | 0.0482 |  |  |  |  |  |
| Lead | 29.3 | 0.0482 |  |  |  |  |  |
| Mercury | 0.0983 | 0.00924 |  |  |  |  |  |
| Nickel | 20.2 | 0.0482 |  |  |  |  |  |
| Selenium | 1.16 | 0.0482 |  |  |  |  |  |
| Silver | 0.42 | 0.0482 |  |  |  |  |  |
| Thallium | 0.365 | 0.0482 |  |  |  |  |  |
| Vanadium | 17.6 | 0.0482 |  |  |  |  |  |
| Zinc | 65.9 | 0.0482 |  |  |  |  |  |

**SUPPLEMENTARY FIGURES**


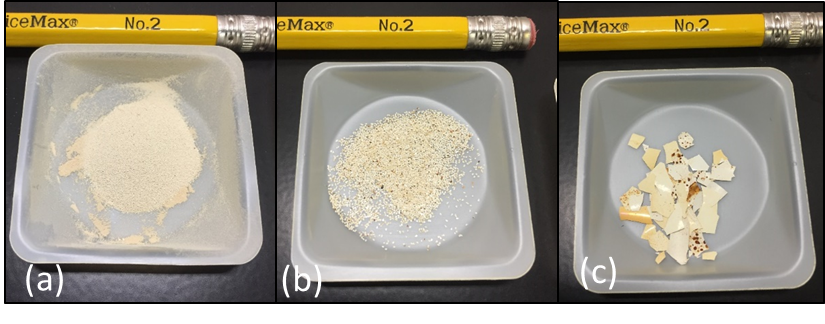


**Fig. S1** PCB-containing PCs sorted to different size classes: a) fine, <0.045 mm; b) medium, 0.250-0.300 mm; c) coarse, 2-5 mm.

**Fig. S2** PCB congener profiles for PCB-containing PCs and for PCB-containing fine PCs added to HSL sediment (HSL+PC_FINE_). Analytes contributing <0.5% to the ΣPCBs concentration were omitted.

**Fig. S3** Average (± 1 standard deviation) ∑PCBs PSAF for HSL sediment amended with fine (HSL+PC_FINE_), medium (HSL+PC_MEDIUM_) or coarse (HSL+PC_COARSE_) PCB-containing fine PCs. The PSAF for HSL was significantly higher than for HSL+PC_FINE_, HSL+PC_MEDIUM_ and for HSL+PC_COARSE_.

**Fig. S4** Average (± 1 standard deviation) PSAF for HSL sediment amended with fine (HSL+PC_FINE_), medium (HSL+PC_MEDIUM_) or coarse (HSL+PC_COARSE_) PCB-containing PCs. Analytes are those detected in all treatments. For all congeners, the PSAF for HSL+PC_FINE_ was significantly higher than those for HSL+PC_MEDIUM_ and for HSL+PC_COARSE_ but the PSAFs for HSL+PC_MEDIUM_ and HSL+PC_COARSE_ were not significantly different.

**Fig. S5** Average (± 1 standard deviation) concentration of PCB congeners in sediment (A) and in PS (B) following different durations of *ex situ* active mixing passive sampling of PCB-containing fine PCs amended to HSL sediment. Analytes contributing <0.5% to the ΣPCBs concentration were omitted. For all congeners, the concentration in PS were not significantly different across treatments.

**Fig. S6** PCB homolog group profiles for PCB-containing PCs and MH sediment.

**Fig. S7** Average (± 1 standard deviation) concentrations of PCB congeners in sediment (A) and in PS (B) for MH sediment. Analytes contributing <0.5% to the ΣPCBs concentration in the sediment were omitted.

**Fig. S8** PCB congener profiles for PCB-containing PCs and MH sediment for PCBs present in PC, i.e., those analytes corresponding to red bars in Fig. 4.

**Fig. S9** Fold increase in concentrations resulting from adding fine size fraction PCB-containing paint to the MH sediment calculated for each analyte using the average concentration for the MH+PC_FINE_ and that for the MH treatment. Black bars shown increase in the sediment concentrations and red bars show increase in the PS following *ex situ* passive sampling following 60 d of mixing. Analytes are PCBs present in PC, i.e., those corresponding to red bars in Fig. 4.

**Fig. S10** Average (± 1 standard deviation) PSAF of PCB congeners absent from PC following *ex situ* passive sampling for the MH and MH+PC_FINE_ treatments. Analytes are those for which the fractional contribution to ∑PCBs in MH sediments was >50 times higher for MH sediment than for PCB-containing PCs. For all congeners, the PSAFs for MH and MH+PC_FINE_ were not significantly different.
